# Clinical scientist in clinical bioinformatics – the under-implemented recommendation of the Topol Review?

**DOI:** 10.1016/j.fhj.2026.100513

**Published:** 2026-02-18

**Authors:** Michal Pruski

**Affiliations:** a School of Health Sciences, The University of Manchester, Manchester, UK; b PIER, Cardiff and Vale UHB, Cardiff, UK

**Keywords:** Clinical informatics, Digital health, Healthcare staff

## Abstract

One of the recommendations of the Topol Review was the ‘expansion of Higher Specialist Scientist Training (HSST) for clinical bioinformaticians’. Freedom of Information requests show that there has been little uptake of bioinformatics trainees, both into the Scientist Training Programme and the HSST. Moreover, there is poor retention of those who completed the training, and evidence of poor appreciation of clinical scientists trained in bioinformatics. Considering that 1) the HSST programme is the only nationally recognised specialist training programme in informatics that allows entry onto an NHS-recognised specialist register, 2) the Scientist Training Programme allows trained scientists to become statutory registered healthcare professionals, 3) organisations receive funding to take up trainees; this under-implementation is hard to understand. It is unclear if NHS organisations are simply unaware of these opportunities, are not able to accept trainees, or do not appreciate the skills of healthcare scientists.

## Introduction

In the UK, we are facing shortages of qualified healthcare staff and there is also a need for skilled informaticians to help with the digital transformation of healthcare. This paper will show that this situation could be alleviated by better use of the clinical science workforce. It first introduces the clinical science profession and their training, particularly in reference to informatics. It then outlines Topol Review recommendations relating to this staff group and discusses their uptake. Finally, it presents some reasons why there might be a low uptake of clinical scientists in the healthcare informatics workforce.

## Clinical scientist training

Clinical scientists are a group of healthcare professionals who fall under the governance of the Health and Care Professions Council (HCPC). While technically once registered, clinical scientists are not committed to one field of practice, their training is designed to prepare them for work primarily within a specialism. Three of these specialisms fall under the broad umbrella of informatics:

• clinical informatics – previously clinical bioinformatics (health informatics).
• clinical bioinformatics genomics – previously clinical bioinformatics (genomics).
• clinical scientific computing – previously clinical bioinformatics (physical sciences).

Genomics bioinformaticians occupy a unique niche, focusing on either human or pathogen genome analysis. Those working in scientific computing and health/clinical informatics operate in a sphere overlapping with other digital healthcare professionals, since they are trained in such areas as implementation science and data analysis.^1^

The standard route for becoming a clinical scientist is via the 3-year graduate-entry Scientist Training Programme (STP).^2^ There is a further 5-year Higher Specialist Scientist Training (HSST) programme available, of similar standard to medical specialist training, which is designed to prepare clinical scientists for consultant posts (NHS band 8c and above), providing specialist, leadership and research training.^3^^,^^4^ Individuals can undertake their HSST training in a different specialism to their STP qualification, but this is probably the exception. Both STP and HSST training is overseen by the National School of Healthcare Science (NSHCS); the same body which oversees the Topol Digital Fellowship Programme.^2^^,^^4^^,^^5^ Health boards/trusts can usually access funding that will provide the STP trainee’s salary (NHS band 6), help with training costs, and cover the cost of the trainee’s master’s degree.^2^ As such, by taking up an STP trainee, a department is contributing to expanding the pool of registered healthcare professionals in the UK while also expanding the informatics workforce. HSST funding is currently at £13,796 a year and additionally also covers the trainee’s doctoral degree fees.^4^ Both STP and HSST trainees need to complete an academic degree and a separate set of work-based competencies assessed by a training supervisor and the NSHCS. Some biomedical scientists are also eligible to undertake HSST training, but this is beyond the scope of this commentary.

While the HCPC maintains the statutory register of clinical scientists, the Academy for Healthcare Science maintains a professional standards accredited register for those who have completed HSST training (or an equivalency portfolio). This Higher Specialist Scientist register currently only has one registrant in genomics and none in the other two informatics specialisms.^6^

Clinical scientists appear to be the only healthcare professional group with a formally recognised specialist programme in informatics, as there is e, no General Medical Council-recognised specialism in informatics. While there are other training programmes that allow healthcare professionals in the UK to complete informatics training, none result in enrolment on a professional specialist register.

## Topol Review recommendations

The need for expanding the clinical scientist workforce to help with the digital transformation was mentioned in the Topol Review^7^:

*‘An attractive career pathway should be developed for bioinformaticians, including expansion of Higher Specialist Scientist Training for clinical bioinformaticians. (G6)’ (pp. 14, 41 & 43).*

*‘For both existing and new roles addressing skills gaps in clinical bioinformatics, digital technologies, AI and robotics, the NHS should develop or expand both educational programmes (for example, the Higher Specialist Scientist Training) and attractive career pathways. (E9)’ (pp. 17 & 81).*

This recommendation appears to have had little tangible impact. Data from 2018–2025 (Freedom of Information requests made to NHS England: FOI-2508-2251562 (2025) and FOI-2402-2067858 NHSE:0796360 (2024)) show that there have been 22 HSST posts advertised in England and Wales for the bioinformatics specialisms, out of which 19 were taken up and 10 trainees left the programme in that time. Of the five health/clinical informatics HSST trainees, two have left. Regarding the STP, during 2018–2025:

• clinical bioinformatics genomics: 114 places were advertised with 107 taken, eight trainees left the programme and 60 were employed in the NHS after completion.
• scientific computing: 59 places were advertised with 46 taken, two trainees left the programme and 15 were employed in the NHS after completion.
• clinical informatics: 33 places were advertised with 29 taken, six trainees left the programme and 12 were employed in the NHS after completion.

Of course, the statistics pertaining to those who left the programme and were employed by the NHS relate to intakes prior to 2018, and those who entered from 2022 have not yet qualified at the time of data gathering, and so could not contribute to staff that stayed in the NHS after qualifying.

In Wales, fewer than five places were commissioned per year for all three specialisms in 2018–2025, for both STP and HSST (Freedom of Information requests made to Health Education and Improvement Wales: FOIDLlB/FOI/21-08-2025 (2025) and DLlB/FOI/02/02/24 (2024)). More specifically, HSST traineeships were only commissioned in 2019 and 2021. The last year for which any STP trainees in health/clinical informatics were offered places was 2020.

## Potential reasons for under-implementation

Such were the concerns regarding retention from informatics STP programmes that a report was commissioned into the future of the NHS clinical bioinformatics workforce. Importantly, the report has shown that only 13% of respondents did not plan a career in the NHS. It illustrated examples of trainees trying to stay in the NHS for as long as possible, despite being sent offers for other work opportunities.^8^ Moreover, the report highlights that since all trainees are graduates, with the majority already having doctoral degrees, they actively made a choice not to pursue an academic career.^8^ Based on the findings of that report, as well as the author’s personal experience, there are three broad reasons for the under-implementation of the HSST.

The first is a simple lack of awareness of HSST or the broader role of clinical scientists within healthcare informatics. This means that healthcare leaders might not be aware of the programme, despite it being mentioned in the Topol Review. Alternatively, they may simply not be aware of the skills of clinical scientists trained in the informatics specialisms (eg, by being mistaken for IT support).^8^ Similarly, poor engagement from digital leaders was also highlighted in relation to the Topol Digital Fellows Programme.^5^ If this is the case, then this commentary might help alleviate this situation by raising awareness.

The second potential reason is a lack of training resources. These include a lack of opportunities to deliver training due to great departmental workloads and ‘constant firefighting’ of problems.^8^ Yet, since many of these trainees already have postgraduate qualifications, after a routine induction, like any other new staff member, they could help ease staffing pressures. Moreover, if organisations can support current clinical staff by paying for postgraduate courses, HSST costs should not be a barrier, since training posts come with additional funding that can be used to eg, back-fill staff time.^4^

Lastly, there might be a simple lack of vacancies. Organisations might simply not have dedicated clinical informatics departments, especially if they are smaller, which perform very different tasks from IT support teams.^8^ Moreover, informatics jobs in NHS organisations are often categorised as administrative,^9^ and not senior enough to warrant HSST training. This means that there simply might not be any opportunities for trainees to go into relevant posts once they have completed the programme. Even for the STP graduates, it is envisaged that after qualification they would generally enter a NHS band 7 job.^10^ This problem might be exacerbated in organisations that require senior digital leadership positions, such as chief clinical informatics officers, to be medical doctors, despite such leadership positions being particularly suited to HSST trainees, who complete leadership and management training as part of the programme.^4^ If there are no exit posts for trainees, then having a trainee might seem pointless to such organisations.

## Discussion

There seems to be little engagement with the clinical scientist informatics professions from the informatics professional bodies. Neither the Federation for Informatics Professionals in Health and Social Care (FedIP) itself, nor any of its member organisations, feed into the Academy for Healthcare Science’s Professional Bodies Council. Conversely, the Institute of Physics and Engineering in Medicine (which encompasses scientific computing), is not a FedIP member. Moreover, while the Academy for Healthcare Sciences is an umbrella organisation allowing all other healthcare science organisations to cooperate, it does not lobby for specific specialisms, which is the task of individual professional bodies. The small number of informatics clinical scientist professionals and their diverse roles make it difficult for these professionals to form interest groups within various informatics professional bodies. A single professional body would potentially be able to alleviate some of the problems relating to the lack of awareness of the programme or lack of recognition of the skillset of clinical scientists.

In all areas of healthcare, it is important that we disseminate skills and knowledge among various staff groups, but also that we have dedicated specialists, such as consultants; the same holds true for informatics. Clinical scientists on their own will not provide the answer to the shortage of digitally trained healthcare staff, and hence opportunities for digitally trained nurses and doctors are important. Yet, under-implementation of the most comprehensive informatics training programme for healthcare professionals represents a significant missed opportunity for digital transformation, as the informatics HSST programmes are designed to produce consultant-level healthcare informaticians.^3^

## Summary

While the STP and HSST programmes provide funding and a nationally established training pathways for developing new healthcare professionals with informatics skills, NHS organisations have under-implemented this opportunity. Considering the mix of technical, leadership and research skills that the trainees develop over the 5-year period, it would seem that HSST graduates would be ideal candidates to drive the digital transformation of the NHS. The way to remedy this, aside of mandating the establishment of clinical scientist consultant posts in informatics departments, remains unclear. Nevertheless, if awareness of the programme is the main problem, then this opinion piece can help alleviate it by providing information and provoking discussion.

## CRediT authorship contribution statement

**Michal Pruski:** Writing – review & editing, Writing – original draft, Project administration, Investigation, Formal analysis, Data curation, Conceptualization.

## Declaration of competing interest

The authors declare the following financial interests/personal relationships which may be considered as potential competing interests: Michal Pruski reports financial support was provided by NHS Wales Health Education and Improvement Wales. Michal Pruski is a senior clinical scientist undertaking the HSST in health/clinical Informatics. If there are other authors, they declare that they have no known competing financial interests or personal relationships that could have appeared to influence the work reported in this paper.
